# Uveitis and Myositis as Immune Complications in Chemorefractory NK/T-Cell Nasal-Type Lymphoma Successfully Treated with Allogeneic Stem-Cell Transplant

**DOI:** 10.1155/2016/7297920

**Published:** 2016-10-11

**Authors:** Maria José Gómez-Crespo, Aránzazu García-Raso, Jose Luis López-Lorenzo, Teresa Villaescusa, María Rodríguez-Pinilla, José Fortes, Cristina Serrano, Salma Machan, Pilar Llamas, Raúl Córdoba

**Affiliations:** ^1^Lymphoma Unit, Department of Hematology, Fundación Jiménez Díaz University Hospital, Health Research Institute IIS-FJD, Madrid, Spain; ^2^Department of Pathology, Fundación Jiménez Díaz University Hospital, Health Research Institute IIS-FJD, Madrid, Spain; ^3^Department of Dermatology, Fundación Jiménez Díaz University Hospital, Health Research Institute IIS-FJD, Madrid, Spain

## Abstract

NK/T-cell lymphomas are a group of clonal proliferations of NK- or, rarely, T-cell types and have peculiar clinicopathologic features. Most common site of involvement is the upper aerodigestive tract (nasal cavity, nasopharynx, paranasal sinuses, and palate). Association of autoimmune paraneoplastic disorders with NK/T-cell lymphomas is not well studied. Our patient was diagnosed with NK/T-cell lymphoma stage IV with skin involvement and treated frontline with CHOEP regimen. While he was under treatment, two immune complications presented: anterior uveitis of autoimmune origin refractory to steroids and myositis in lower limbs muscles. Autologous transplantation was rejected due to confirmed early relapse after first-line treatment, and the patient received second-line treatment according to the SMILE scheme, reaching complete response after four cycles. The patient underwent allogeneic transplantation and at the time of manuscript preparation is alive despite multiple complications. The disease should be suspected in patients with rhinitis or recurrent sinusitis, and early biopsy is recommended for all patients to avoid a delay in diagnosis. Our patient also presented symptoms of disease progression after first-line treatment, representing a paraneoplastic process, a very rare phenomenon in T-type lymphomas. This case is novel for the appearance of an inflammatory myositis, a histologically verified paraneoplastic phenomenon that responded to treatment for lymphoma.

## 1. Background

NK/T-cell lymphomas are a group of clonal proliferations of NK- or, rarely, T-cell types and have peculiar clinicopathologic features. The most common site of involvement is the upper aerodigestive tract, including the nasal cavity, nasopharynx, paranasal sinuses, and palate [1]. More rarely, extranodal NK/T-cell lymphomas can present in other sites such as the skin, testis, lung, or gastrointestinal tract [2]. The World Health Organization (WHO) has subcategorized NK/T-cell lymphoma into extranodal NK/T-cell lymphoma, nasal extranodal NK/T-cell lymphoma (ENK/T-N), and extranodal NK/T-cell lymphoma nasal-type (ENK/T-NT) [1, 3, 4]. Extranodal NK/T-cell lymphomas have low survival rates and poor response to treatment [5]. Nasal-type lymphoma is clinically less aggressive, is more localized, and has better prognosis [6]. The term ENK/T-NT was adopted by the WHO in replacement of angiocentric lymphoma. The ENK/T-NT category accounts for less than 2% of non-Hodgkin's lymphomas in Europe and North America but is more frequent in Asia and South and Central America [7]. Histopathologically, this aggressive disease is characterized by a positive Epstein-Barr virus (EBV) [8], atypical lymphoid cytotoxic infiltrate, extensive vascular destruction, and prominent tissue necrosis [2]. Some groups have reported that viral load is a useful predictor for monitoring response to treatment [4]. The association of malignancy with paraneoplastic events is well known, and sometimes the clinical and radiologic features can mimic cellulitis, panniculitis, or fasciitis [2, 6, 9].

## 2. Case Presentation

We report a very rare case of stage IV nasal-type NK/T-cell lymphoma with metastatic lesions in the right leg mimicking cellulitis on initial clinical-radiologic diagnostic workups that included radiology studies. To our knowledge, this is the first report of cutaneous involvement of nasal-type NK/T-cell lymphoma presenting as myositis of the leg.

**Figure 1:**
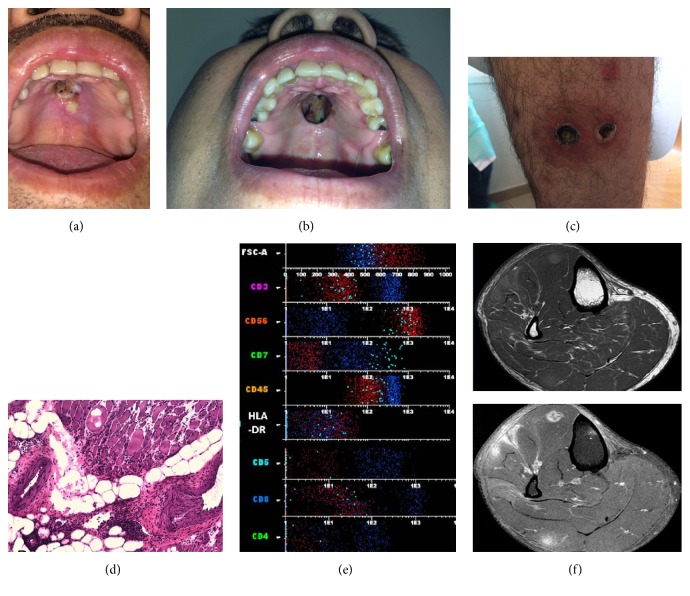
(a-b) Nasal lymphoma showing an initial ulcer that perforated into the oral cavity, opening a passage between the oral cavity and the nasal cavity. (c) Cutaneous manifestation of nasal-type extranodal NK/T-cell lymphoma. (d) Skeletal muscle fascicles revealing markedly distorted overall architecture with inflammatory interstitial infiltrate and endo and perimysial expansion. Perivascular lymphocytic inflammatory cluster with no signs of vasculitis. Myopathic changes suggestive of inflammatory myopathy. EBER negative. (e) Flow cytometry analysis of vitreous humor specimen: analysis is performed on cells consistent with leukocytes by FSC versus SSC and CD45. Flow cytometry demonstrates an aberrant NK-cell population (highlighted in red) with the following immunophenotype: CD3s−, CD56 (bright), CD7−, CD45+, HLA-DR−, CD5−, CD8−, and CD4− (see complete immunophenotype in text). Cells highlighted in cyan show a minor population of not aberrant NK cells and cells in red show reactive T cells. (f) MRI of the lower limbs revealing myositis infectious between the extensor digitorum longus and peroneus longus.

A 56-year-old Ecuadorian man was referred to our department with a 7-year history of bilateral nasal respiratory insufficiency resistant to multiple treatments (antibiotics, steroids, etc.). In August 2013, anterior septal perforation (11 × 12 mm) was observed using computed tomography (CT). Nasal biopsy was proposed but the patient was lost to follow-up. In September 2014, the patient presented to the dermatology department with complaints of progressive skin lesions on the trunk, legs, and glands that consisted of subcutaneous nodules tending to ulcerate. Palate perforation was also observed upon examination. Biopsy of the lesions and a CT scan were performed. A few days later, the patient reported to the emergency department due to ulcers on the palate and fever reaching 39°C. Septum and palate perforation were observed on CT images (Figures 1(a) and 1(b)). The results of laboratory testing on admission were white cell count of 6.87 × 10^3^/*μ*L, hemoglobin of 14.8 g/dL, platelet count of 182 × 10^3^/*μ*L, total bilirubin 0.6 mg/dL (normal range 0.3–1.2 mg/dL), alkaline phosphatase 104 U/L (45–129 U/L), aspartate aminotransferase 34 U/L (0–34 U/L), albumin 3.6 g/dL (3.2–4.8 g/dL), *γ*-glutamyl transferase 73 U/L (0–73 U/L), lactate dehydrogenase (LDH) 587 U/L (230–460 U/L), creatinine 0.9 mg/dL (0.7–1.3 mg/dL), triglyceride levels 227 mg/dL (<200 mg/dL), ferritin 768 ng/mL (20–250 ng/mL), and C-reactive protein 16.07 mg/dL (0–0.5 mg/dL). Blood cultures were carried out with negative results.

Additionally, on physical examination the patient presented necrotic, ulcerated, cutaneous nodules (Figure 1(c)) located on trunk and extremities. Mucormycosis and other possible infections were ruled out in differential diagnosis, and the patient was admitted for study. Cutaneous tissue culture evidenced growth of* Staphylococcus aureus*. The skin biopsy showed infiltration by atypical medium-to-large size lymphoid cells with epidermotropism, angiocentricity and angiodestruction with large areas of necrosis. The tumoral cells exhibited weak expression of CD3, TIA1, CD2, and CD56. The neoplastic cells did not express CD5, CD7, CD4, CD8, CD20, or TCRBF1. Furthermore,* in situ* hybridization for Epstein-Barr virus (EBV) encoded RNAs was positive. The overall features were consistent with extranodal nasal-type NK/T-cell lymphoma. During admission, the patient was referred to the hematology department, where he began CHOEP induction therapy, receiving six cycles of the treatment and exhibiting improvement of skin lesions without associated complications, except blurred vision during the second cycle and left-eye optic neuritis during the third session. The patient was assessed by the ophthalmology department and was diagnosed as having anterior uveitis with probable herpetic origin. Treatment with valacyclovir led to a poor outcome; however the anterior uveitis improved with steroids but without complete resolution.

While he was waiting for consolidation therapy with autologous stem-cell transplantation, he developed very painful skin lesions that consisted of cutaneous infiltration by lymphoma cells and suffered again of blurred vision. New ophthalmic assessment discovered a posterior uveitis. Analysis by flow cytometry immunophenotype of vitrectomy sample (vitreous humor) demonstrated elevated leukocyte count with an aberrant NK-cell population comprising approximately 25% of the cells. This atypical population was within the lymphoid cell gate, with higher forward scatter properties as the residual normal lymphoid cells. The aberrant NK-cell population expressed CD45, CD56 (bright), and CD2 and was negative for CD7, CD3s, CD3cy, TCR alpha-beta CD4, CD5, CD8, TCR gamma-delta, CD16, CD10, CD14, CD19, CD20, CD34, and HLA-DR (Figure 1(e)). These findings are consistent with the infiltration by NK lymphoma. Furthermore, painful, palpable subcutaneous nodules appeared on the right leg. Magnetic resonance imaging (MRI) showed diffuse soft tissue infiltration and subcutaneous edema (Figure 1(f)). These findings were also suggestive of myositis. A muscle biopsy of the right leg was subsequently performed and revealed a multifocal, chronic inflammatory infiltrate of small lymphocytes with scattered muscle fiber necrosis, consistent with a diagnosis of polymyositis (Figure 1(d)), with no evidence of lymphoma. After this episode of disease recurrence, the patient received second-line treatment according to the SMILE scheme [14]. He achieved complete response after four cycles and underwent allogeneic transplantation on June 26, 2015, from a HLA identical sibling donor. After the transplantation, the patient developed several complications, including severe mucositis with vomiting, malnutrition, and catheter tunnel infection, biopsy-confirmed acute cutaneous and gastrointestinal graft versus host disease (GVHD), with no histologic confirmation of CMV reactivation.

## 3. Discussion

The most common hematologic malignancies associated with dermatomyositis/myositis are B-cell lymphomas [15]. Extranodal NK/T-cell nasal lymphoma is rarely associated with skin lesions mimicking inflammatory or reactive disorders with a similar histopathologic pattern. Muscle involvement is also an uncommon finding. Very few cases have been reported in the literature of T- and NK-cell lymphomas with muscular involvement with initial presentation of muscle swelling or weakness mimicking myositis [2, 10, 11, 16–20]. Moreover, many neoplasms are associated with paraneoplastic phenomena as a side effect of the production of biologically active hormones, growth factors, cytokines, antibodies, and so forth, by the tumor cells. These phenomena are sometimes the first sign of the disease and involve mandatory rule out metastases, infection, vascular processes, and so forth. In the case of nasal NK/T-cell lymphoma, some paraneoplastic phenomena have been described as pyoderma gangrenosum or paraneoplastic pemphigus, even lesions simulating dermatomyositis/myositis [10, 15]. When reviewing the literature, we found five cases of extranodal nasal-type NK/T-cell lymphoma associated with autoimmune phenomena or inflammatory panniculitis with a maximum survival of seven months [2, 9–13]. None of the studies found described myositis or uveitis as paraneoplastic phenomena (Table 1).

Our patient presented paraneoplastic lesions, inflammatory myositis, and anterior uveitis, not described in the literature; these lesions resolved fully after targeted therapy for lymphoma. In addition, we suspect that the anterior uveitis also had an immune origin, as uveitis did not respond to treatment with valacyclovir and improved with chemotherapy.

In conclusion, we present a case of stage IV nasal-type NK/T-cell lymphoma. This entity has a very low incidence in our environment and, despite its sensitivity to radiotherapy, prognosis is poor. The disease should be suspected in patients with rhinitis or recurrent sinusitis, and early biopsy is recommended for all patients to avoid a delay in diagnosis. Our patient also presented symptoms of disease progression after first-line treatment, representing a paraneoplastic process, a very rare phenomenon in T-type lymphomas. This case is novel for the appearance of an inflammatory myositis, a histologically verified paraneoplastic phenomenon that responded to treatment for lymphoma. This case is also noteworthy because the patient is still alive nine months after allogeneic bone marrow transplantation without evidence of relapse, so this strategy should be the treatment of choice in fit patients with available donor [21, 22].

## Figures and Tables

**Table 1:** Reported cases with autoimmune complications.

Author	Age	Sex	Location	Previous symptoms	Autoimmune complications	Time to diagnosis	Treatment	Survival	Cause of death
Park et al. [10]	40	W	Skin	High fever, proximal muscle weakness, multiple skin plaques with bullae and serous discharge	Dermatomyositis and hemophagocytic syndrome	24 months	CHOP → steroid, antibiotics, gamma globulin, oral cyclosporin	7 days	Hepatic failure, renal failure, pancytopenia, massive pleural effusion
Kim et al. [2]	64	M	Skin	Painful swelling and redness of the left upper arm	Cellulitis or fasciitis	5 months	Antibiotics → L-asparagine chemotherapy	n.r.	n.r.
Chan et al. [11]	68	W	Muscle	Forearm swelling and bilateral facial swelling	Polymyositis	4-5 weeks	Prednisolone	Few days	Fulminant hemophagocytic syndrome
Spadigam et al. [12]	49	M	Skin	Intermittent fever, nasal stuffiness, epistaxis and hemifacial pain, nasolabial lesion	Inflammatory myofibroblast	3 months	Surgery	2 weeks	Postoperative complications
Fei et al. [13]	83	W	Lung	Skin ulceration and intermittent fever	Panniculitis	12 months	Antibiotics → patient refused treatment	35 days	Multiorgan failure
Chow et al. [9]	27	W	Skin	Intermittent fever, occasional night sweats, nasal congestion and hoarseness, skin nodules	Panniculitis or sarcoidosis	3 months	Antiviral/antibiotic → CHOP → ICE	7 months	Secondary hemophagocytic syndrome
Current	56	M	Skin	Bilateral nasal respiratory insufficiency, anterior septal perforation, skin lesions	Myositis and uveitis	13 months	CHOP → SMILE → BMT	30 months	Alive
